# The effect of genetically predicted higher adulthood body mass index on blood pressure traits: A protocol for a systematic review and meta-analysis of Mendelian randomisation studies.

**DOI:** 10.12688/wellcomeopenres.26288.1

**Published:** 2026-04-30

**Authors:** Winfred Gatua, Ge Chen, Farhad Shokraneh, Yi Liu, Deborah A Lawlor, Julian PT Higgins, Tom.R. Gaunt

**Affiliations:** 1 MRC Integrative Epidemiology Unit, University of Bristol Medical School, Bristol, England, BS1 5DS, UK; 2 Population Health Sciences, University of Bristol Medical School, Bristol, England, BS1 5DS, UK; 3 Bristol Dental School, University of Bristol Dental Hospital, Bristol, England, UK; 4 Department of Clinical and Biological Sciences, University of Exeter, Exeter, England, UK; 5 Centre for Academic Primary Care, University of Bristol Medical School, Bristol, England, BS8 2PS, UK

**Keywords:** Body mass index; diastolic blood pressure; systolic blood pressure; hypertension; Mendelian randomisation; randomised controlled trials; risk of bias

## Abstract

**Background:**

Mendelian randomisation (MR) studies have been used to investigate whether higher body mass index causally influences blood pressure outcomes. However, conclusions vary across studies, potentially reflecting differences in risk of bias, populations and analytical methods. This systematic review aims to synthesise MR evidence on the effect of variation in adulthood body mass index (BMI) on blood pressure outcomes.

**Methods:**

We will search three databases (Embase, MEDLINE and Science Citation Index). Two reviewers will independently screen titles and abstracts and full texts for inclusion according to eligibility criteria. Data will be extracted into predefined tables using Microsoft Excel. Risk of bias and certainty of evidence will be assessed using modified risk-of-bias tools and Grading of Recommendations, Assessment, Development, and Evaluation approach (GRADE), respectively. The evidence will then be synthesised and presented in figures and tables, as appropriate. The reporting of this systematic review will follow the PRISMA-2020 guidelines.

**Anticipated findings:**

By comparing methods and results and potentially meta-analysing results, we will be able to precisely estimate the magnitude and likelihood of the causal effect of adulthood BMI on blood pressure. As well as examining whether the findings are consistent across studies, we will also determine risk of bias for each exposure-outcome pair. We will establish sources of between study heterogeneity. In subsequent work, this systematic review will be triangulated with evidence from randomised controlled trials.

## Plain Language summary

Estimating if a change in a risk factor causes disease risk is vital for public health. Mendelian randomisation (MR) uses natural genetic variation to explore cause-and-effect relationships. Randomised controlled trials can be used to test the effect of the target of the RCT intervention (e.g. BMI in a weight-loss trial) on an outcome (e.g. blood pressure). MR has been used to study the effect of variation in BMI on blood pressure (BP). The conclusions vary across studies, possibly due to study differences and bias. We do not know what causes differences between studies and their impact on the true causal effect of BMI on BP. Comparing results of a risk factor such as BMI on an outcome such as BP between MR and RCTs can boost our confidence in conclusions. But it can be problematic because we do not have a good tool to judge bias in MR studies.

In this review, we will gather all peer-reviewed MR studies of the effect of variation in BMI on BP measures. We will search Embase, MEDLINE and Scientific Citation Index from their start up to 4 ^th^ March 2026. Two reviewers will independently screen all studies by title and abstract, and then full text, to decide which ones meet our inclusion criteria.

We will extract data into predefined tables. With expert support, we will adopt proposed guidelines for assessing risk-of-bias in MR studies. We will rate quality of evidence using Grading of Recommendations, Assessment, Development, and Evaluation approach.

This review will summarize MR evidence on how BMI affects BP. It will highlight how bias and study differences may shape the findings. We expect the results to show higher BMI increases BP. We will identify reasons why some MR studies reach different conclusions. These findings will inform a future project comparing MR and RCT evidence.

## Background

Mendelian randomisation (MR) refers to the use of genetic variants to strengthen causal inference regarding the effect of exposures (such as body mass index (BMI), as planned in this review) on outcomes, such as blood pressure. ^1^ Most MR studies use instrumental variable (IV) analyses, ^2^ which involves identifying genetic variants that are statistically strongly associated with the exposure as IVs to estimate its effect of the exposure on one or more outcomes. This approach requires that key assumptions of IV analyses are met. Table 1A summarises these assumptions below, including how they may be violated in MR analyses and how these violations may be explored and/or mitigated.

**Table 1.** IV assumptions and source of bias A, MR-IV and B, RCT-IV.

	Assumption	Potential bias
**A**	IV is strongly statistically associated with the exposure in the relevant population (Relevance).	IV is not statistically strongly associated with the exposure in the relevant population. Bias can occur if the genetic IV, which usually consists of a pooled set of all genome-wide significant single nucleotide polymorphisms (SNPs) from large GWAS in a relevant population, is not strongly associated with the exposure. The strength of the mean F-statistic of association with the exposure across all independent SNPs is used to suggest instrument strength. Weak IVs can result in bias towards the null in two sample MR, and away from the null in one sample MR. If the genetic IV is not relevant to the population who experience the outcome, for example if the GWAS data used to select IVs is only available for women and men combined but the study is looking at sex specific outcomes or where pregnancy and perinatal outcomes are explored but the genetic instruments are from GWAS of men and non-pregnant women, there can be bias in either direction. Bias is mitigated by trying to obtain strong instruments and using data from the relevant population if possible, and if not, testing that the genetic IV associates with the exposure in the relevant population in the same direction and with similar magnitude as in the GWAS from which the SNPs for the IV were selected.
	There are no common causes of IV and outcome (Independence).	The common confounders of conventional observational studies such as socioeconomic status cannot change the genetic variants and therefore it is unlikely to violate this assumption. Population stratification, assortative mating and dynastic effects can violate this assumption, introducing confounding to genetic studies. But population stratification is often mitigated by restricting analysis to the same population and adjusting for principal components. Family based studies can be used to avoid bias by population stratification, assortative mating and dynastic effects.
	IV is not associated with the outcome other than through its association with the exposure (Exclusion restriction).	Violation of the exclusion restriction is likely the main source of bias. Horizontal pleiotropy, a phenomenon where genetic IV influences the outcome not through the exposure of interest could violate this assumption. When comparing multiple IVs to the tune of 100s, we may capture other mechanisms from the IV to the outcome other than through the exposure.
**B**	IV is strongly associated with the exposure in the relevant population (Relevance).	IV is not associated with the exposure in the relevant population. Weak instrument bias might be a potential key source of bias in this use of IVs. In RCT IV analysis, assignment to the intervention must meaningfully change the intermediate/exposure (e.g. BMI). If the intervention only minimally changes the exposure, this will lead to bias. If participants do not follow their assigned intervention (non-adherence), this would introduce bias.
	There are no common causes of IV and outcome (Independence).	In well-conducted RCTs, it is unlikely that the IV will be related to confounders. In RCT IV analyses, this assumption is expected to hold since randomisation distributes confounders equally between groups. Nevertheless, chance imbalances in small trials could bias the results. Selection bias post-randomisation (e.g. differential loss to follow-up) can re-introduce associations between the IV and the confounders. The use of appropriate randomisation procedures, adequate sample sizes, and minimizing loss to follow-up could help mitigate any possible confounding.
	IV is not associated with the outcome other than via its association with the exposure (Exclusion restriction).	Violation of the exclusion restriction assumption is likely the main source of bias. This includes any effects of the intervention on the outcome unrelated to the exposure, such as a drug intended to reduce BMI also directly influencing blood pressure through other pathways. Any behavioural change triggered by participating in the trial could also violate this assumption. Restricting the analyses to single-mechanism interventions and use of interventions that specifically target the exposure as well as performing some sensitivity analyses where possible could help mitigate this. However, the mechanisms through which this violation occurs will likely be different from those in MR IV analyses.

Randomised controlled trials (RCTs) test the effect of an intervention on health outcomes. ^3– 5^ RCTs can also be used to test the effect of an exposure, such as change on BMI, on an outcome such as blood pressure. This is possible if the exposure is a target of the intervention; for example, if the RCT is testing the effect of new BMI lowering medications, and the RCT estimates the effect on both the target (BMI) and the outcome of interest (e.g. blood pressure). When used to test such an effect we also use IV analyses, with the IV being whether someone is randomised to intervention or comparison. Using RCT IV analyses in this way also relies on the key IV assumptions; Table 1B summarises these assumptions and their potential violations in this RCT setting.

Triangulation of epidemiological evidence refers to the integration of evidence from different methods that have different and unrelated key sources of bias and address the same causal aim or question. ^6^ If the results are consistent across these different methods with different key biases, then confidence increases in that consistent result reflecting the true causal effect, as we would expect different biases to generate different results. Several triangulation studies have triangulated MR IV and RCT IV evidence to improve causal inference. For example, one study used MR to test the effect of variation in circulating (25[OH]D), the active component of vitamin D, and of variation in calcium levels on birth weight and compared those results with results using IV analyses in RCTs with data extracted from systematic reviews of RCTs of vitamin D and calcium supplements, respectively. The MR results, including sensitivity analyses suggested no effect of (25[OH]D) on birth weight and that calcium possibly had a weak effect on increasing birth weight that was driven by horizontal pleiotropy bias. Using IV analyses in vitamin D supplement RCTs suggested a weak increase in birthweight whereas the analyses of calcium supplement RCTs suggested a marked increase in birthweight which authors described as due to risk of bias. In another study comparing results from MR, RCT IV and conventional multivariate regression, consistent results were found of lower systolic blood pressure reducing the risk of coronary heart disease once the length of time with lower blood pressure was taken into account. ^7^ In addition, a recent framework for triangulation using a quantitative (rather than the more common qualitative) approach demonstrated the consistent null effect of variation in beta-carotene on cardiovascular disease between MR IV, RCTs and conventional multivariable regression analyses. ^8^ Although the key sources of bias are related to IV assumptions in both of these methods, the source of that bias differs between IV analyses in MR and those in RCTs (see Table 1 and ^6– 9^).

Triangulating MR results with RCT evidence and other methods to obtain valid quantitative causal estimates is potentially valuable, but currently limited by the lack of peer-reviewed risk of bias assessments of MR studies.

This systematic review of MR studies on the effect of BMI on blood pressure outcomes forms part of a larger project aimed at further developing triangulation methods that integrate results from IV analyses applied to in both MR and RCTs and explore the value and interpretation of these using qualitative and quantitative integration of results. The aim of this review is to systematically identify the effect of variation in BMI on blood pressure using MR and to explore the effect of risk of bias in these MR studies and between-study heterogeneity.

### Review objectives

1. To systematically identify and select MR studies estimating the causal effect of adulthood BMI on diastolic and systolic blood pressure and hypertension.
2. To assess the risk of bias of selected studies for each of the exposure/outcome combinations.
3. To meta-analyse causal effect estimates of variation in BMI on each outcome, where sufficient data are available.
4. To assess between-study heterogeneity and, where present, to explore the potential role of study characteristics (e.g. exposure instruments, outcome measures, and risk of bias) on between study heterogeneity where data allow.

## Materials and methods

### Data sources

We will perform a comprehensive literature search of the following databases Embase, MEDLINE and Science Citation Index, to identify peer-reviewed research articles investigating the causal effect of adulthood body mass index on blood pressure traits using Mendelian randomisation. The database search will be supplemented by scanning the reference lists of the included studies.

### Eligibility criteria

Inclusion criteria

We will include only studies that use a Mendelian randomization design. Studies will only be included if they are based on adult humans (aged 18 years or older). The exposure must be adulthood body mass index (BMI). The outcome must include at least one of diastolic blood pressure (DBP), systolic blood pressure (SBP), essential/primary hypertension, arterial hypertension.

Exclusion criteria

We will exclude reviews, commentaries, conference abstracts, letters to editors, editorials, perspectives, opinions and non-English publications.

### Search strategy

The search terms have been developed iteratively, incorporating expert knowledge and guidance from information specialists, as shown in Table 2.

**Table 2.** Search terms for Mendelian randomisation studies.

Search terms	
1	exp Body Mass Index/ or body mass index.mp. or body mass index.tw,kw. or exp obesity/ or obesity.tw,kw. or visceral obesity.mp. or exp overweight/ or overweight.tw,kw. or adiposity/ or adiposity.tw,kw. or visceral adiposity.mp. or BMI.tw,kw.
2	high blood pressure.mp. or high blood pressure.tw,kw. or exp Hypertension/ or hypertension.tw,kw. or hypertensive.mp. or hypertensive.tw,kw. or systolic hypertension.mp. or systolic hypertension.tw,kw. or exp Blood Pressure/or systolic blood pressure.mp. or systolic blood pressure.tw,kw. or diastolic blood pressure.mp. or diastolic blood pressure.tw,kw. or Arterial hypertension.mp.
3	exp Mendelian Randomization Analysis/ or Mendelian randomization.mp. or mendelian randomisation.mp or mendelian randomi#ation.tw,kw. or mendelian randomi#ation analysis.tw,kw.
1 and 2 and 3	Combined search terms

### Screening and selection

We will upload all search results from the three databases to EndNote 21. ^10^ We will detect and remove duplicated records using the EndNote 21 “Find duplicates” function. ^11^ Two primary reviewers (Winfred Gatua and Ge Chen) will independently screen the titles and abstracts of articles against the eligibility criteria using Rayyan software.

The full text of articles from the previous step will be sourced before being independently evaluated against the inclusion and exclusion criteria by both primary reviewers. These independent reviewers will then meet to discuss each paper to determine whether it should be included in the systematic review. Any conflicts or disagreements will be sent to the third reviewer (Tom Gaunt) who will independently assess the article(s) for inclusion, consider the assessments of the two primary reviewers, and make a final decision.

### Data collection

Data items

We will extract the following data items:

- Study and author information
  ○ Title (free text)
  ○ Publication year (numeric)
  ○ First author last name (free text)
  ○ Study aim (free text)
- Methods
  ○ MR study design (categorical; choose all that apply)
    • One-sample MR, Two-sample MR, Multivariable MR, MR-PheWAS, factorial MR, Bidirectional MR, Nonlinear MR and Within-family MR
  ○ Other methodological features
    • Was sample overlap addressed? (yes/no/unclear)
    • Was there population restriction? (yes/no; if yes, specify)
  ○ Exposure
    • Exact exposure name (free text)
    • Exposure GWAS sample size – total (numeric)
    • Exposure GWAS sample size – per sex (if stratified) (numeric)
    • Age-range or mean age of those in Exposure GWAS
    • Sex composition (categorical: female, male, both, not recorded)
    • Study/consortium source for Exposure GWAS (e.g. Locke 2015 BMI) (free text)
    • Population or ancestry (categorical: EUR, AFR, EAS, HIS, MIXED)
    • Exposure measurement units (1 SD, 1 kg/m^2, SD log of BMI)
  ○ Outcome
    • Exact outcome name (categorical: DBP, SBP, hypertension)
    • Outcome GWAS sample size – total (numeric)
    • Outcome GWAS sample size – per sex (if stratified) (numeric)
    • Case/control number (numeric, if available)
    • Study/consortium name (e.g. FinnGen) (free text)
    • Age-range or mean age of those included in the Outcome GWAS
    • Sex composition (categorical: female, male, both, Not recorded)
    • Population or ancestry (categorical: EUR, AFR, EAS, HIS, MIXED)
    • Proportion of people on blood pressure lowering medication (numeric % or not recorded)
    • Antihypertensive use adjustment in Outcome GWAS (free text)
    • Outcome measurement units (mm Hg, SD)
  ○ Selection of instrument variables
    • Description of the population from which the instrumental variables were obtained (free text)
    • Details of any covariates included in the model to adjust genetic variant-exposure estimates and standard errors (free text)
    • P-value threshold for selecting the genetic instruments (numeric e.g., 5e-8)
    • LD-clumping threshold used (numeric e.g. r^2 < 0.001)
    • Number of instrumental variables (numeric)
    • Instrument type (categorical: single SNP, multiple SNPs, weighted GRS, unweighted GRS)
    • Is instrument strength reported? (yes/no)
    • If yes:
      □ mean/median F-statistic (numeric)
      □ Variance explained (R^2)(numeric)
  ○ Statistical methods
  ○ What are the causal effect estimation methods used? (categorical; choose all that apply)
    • IVW, IV estimator, Weighted median, Weighted mode, Simple median, Wald ratio, 2SLS, MR-PRESSO, LASSO MR, MR GXE, MR GENIUS, MR-Egger
  ○ Were sensitivity and any pleiotropy analyses performed? (yes/no)
    • MR-Egger intercept test performed (yes/no)
    • Cochran’s Q statistic for heterogeneity (yes/no)
    • MR-PRESSO outlier test (yes/no)
    • Leave-one-out analysis (yes/no)
  ○ Was multiple testing correction applied? (yes/no)
    • If yes:
    • What is the type of correction? (categorical: Bonferroni, FDR, Holm, Hochberg)
- Results
  ○ Extract results for each MR method reported in a new row
  ○ Extract the same data for all the sensitivity methods
  ○ MR effect estimates
    • Effect estimate type (categorical: odds ratio, hazard ratio, risk ratio, risk difference, beta)
    • Effect estimate value (numeric)
    • Standard error (numeric)
    • Lower confidence interval (CI) (numeric)
    • Upper CI (numeric)
    • P-value (numeric)
    • Adjusted P-value (numeric)
    • Units per increase in BMI (free text) (e.g. per 1 SD BMI, per 1 kg/m ^2^ of BMI)

We will develop and pilot a data extraction form with all the above data items prior to performing data extraction. The two primary reviewers (WG and GC) will independently extract the data from the included papers into predefined tables using Microsoft Excel. They will meet to discuss and resolve any inconsistencies. The third reviewer (TG) will resolve any discrepancies that cannot be resolved by the primary reviewers.

Risk of bias assessment

There are no established tools for assessing risk of bias in Mendelian randomisation studies. However, we will adopt the guidelines proposed by Mamluk et al 2021 ^12^ alongside the Risk Of Bias in Non-randomised Studies of Exposure effects (ROBINS-E) ^13^ and Strengthening The Reporting of Observational studies in Epidemiology using Mendelian randomisation (STROBE-MR) guidelines, ^14^ with support from our collaborator Professor Julian Higgins (University of Bristol). Two primary reviewers (WG and GC) will independently perform risk-of-bias assessment and discuss them with other members of the review team and collaborators.

Reporting of risk of bias assessment

We will use the risk-of-bias visualisation (robVis) tool ^15^ to visualise the various bias domains and overall bias for every exposure-outcome pair.

Certainty of evidence

We will implement the Grading of Recommendations, Assessment, Development and Evaluations (GRADE) guidelines ^16^ to assess certainty of evidence. The GRADE system examines five domains: limitations in the design or execution of the studies, inconsistency, indirectness, imprecision and dissemination bias.

### Data synthesis strategy

Two independent reviewers (WG and GC) will group the data into the respective one sample MR, two sample MR and MR-Phenome-wide analysis study designs. We will manually review the data for overlap. If more than one study uses overlapping genetic instruments, exposure and outcome data, we will exclude the less powered study from the meta-analysis. If there is evidence of minimal overlap either on exposure or outcome data, we will perform sensitivity analyses, where possible.

Where possible for each BMI-outcome (DBP, SBP, hypertension) we will pool studies using a fixed effect meta-analysis in metafor package ^17^ in R version 4.5.2. Our reason for using a fixed effect meta-analysis is because all effect estimates in an MR setting estimate the same effect for the same exposure-outcome pair. We will perform a narrative synthesis for any BMI-blood pressure where a quantitative meta-analysis is not possible.

We will use the inconsistency metric (I ^2^) to assess the extent to which results are consistent across studies. Additionally, we will conduct subgroup analysis based on the population (ancestry), study quality, MR method used to estimate the causal effect, sex and number of instrumental variables where sufficient data is available to explore sources of heterogeneity.

### Patient and Public involvement

Patients and members of the public will not be involved in the design or conduct of the study as this is a protocol for a systematic review.

### Study status

The study is currently at the title-and-abstract screening stage.

### Protocol and registration

The final summary protocol has been registered in Prospero [registration number: CRD420261306158, ( https://www.crd.york.ac.uk/PROSPERO/view/CRD420261306158 ^18^)] on 20 ^th^ March 2026.

## Ethics and dissemination

This systematic review will not require ethical approval as we will analyse publicly available literature. We will disseminate the findings of this systematic review to diverse stakeholders. Specifically, we will submit this work to a peer-reviewed journal and present the findings to a relevant epidemiology conference.

## Collaborators

Dr Farhad Shokraneh, Centre for Academic Primary Care, Bristol Medical School, University of Bristol.

Dr Yi Liu, MRC Integrative Epidemiology Unit, Population Health Sciences, Bristol Medical School, University of Bristol.

Prof Deborah A Lawlor, MRC Integrative Epidemiology Unit, Population Health Sciences, Bristol Medical School, University of Bristol.

Prof Julian PT Higgins, MRC Integrative Epidemiology Unit, Population Health Sciences, Bristol Medical School, University of Bristol.

## Data Availability

We will not generate any new data from this systematic review. However, all data extracted from the papers included in the review will be made available as extended data alongside the publication of the review. All the code used in this review will be made available via GitHub. The reporting of this study protocol has followed the PRISMA-P 2015 reporting guidelines. ^19^ Zenodo: PRISMA-P checklist for ‘The effect of genetically predicted higher adulthood body mass index on blood pressure traits: A protocol for a systematic review and meta-analysis of Mendelian randomisation studies’, https://doi.org/10.5281/zenodo.19063219. ^20^ Data are available under the terms of the Creative Commons Zero "No rights reserved" data waiver (CC0 1.0 Public domain dedication).
