# Supplementary material for: The effect of genetically predicted higher adulthood body mass index on blood pressure traits: A protocol for a systematic review and meta-analysis of Mendelian randomisation studies
Source: Wellcome Open Res. 2026 Apr 30;11:250. [Version 1] doi: 10.12688/wellcomeopenres.26288.1 (PMC13555081; doi:10.12688/wellcomeopenres.26288.1)
Supplement: Peer review history

# Reviewer report: Reviewer response for version 1

**DOI:** 10.21956/wellcomeopenres.28950.r155372

**Published:** 2026-07-29

**Authors:** Sander W. van der Laan

**Affiliations:** 1 Department of Genome Sciences, University of Virginia (Ringgold ID: 2358), Charlottesville, Virginia, USA

The team by Gatua et al. will execute a meta-analysis of MR and a systematic review to assess the effect of variation in (adult) BMI on blood pressure outcomes. 

 This is a well thought-through protocol. I only have a few comments to be addressed.

 Many studies were included in meta-analyses of GWAS on blood pressure phenotypes and BMI, and many of those studies did so in both. In other words, there will be sample overlap between the two outcomes (BMI and blood pressure).

 Additionally, the authors assume a fixed-effects model stating "...all effect estimates in an MR setting estimate the same effect for the same exposure-outcome pair...". However, the between-study heterogeneity is unlikely to be zero: studies contributing to those GWAS will have been treated for blood pressure, and will have other risk factors confounding the phenotypes (tested in the GWAS). 

 So:

 - How will the authors tackle the overlapping samples between (meta)GWAS?

 - Why not use a random-effects model when we know the heterogeneity will not be zero?

Is the study design appropriate for the research question?

Yes

Is the rationale for, and objectives of, the study clearly described?

Yes

Are sufficient details of the methods provided to allow replication by others?

Yes

Are the datasets clearly presented in a useable and accessible format?

Yes

Reviewer Expertise:

Cardiovascular genetics in the field of atherosclerosis.

I confirm that I have read this submission and believe that I have an appropriate level of expertise to confirm that it is of an acceptable scientific standard.

# Reviewer report: Reviewer response for version 1

**DOI:** 10.21956/wellcomeopenres.28950.r157764

**Published:** 2026-06-25

**Authors:** Amand Floriaan Schmidt

**Affiliations:** 1 University College London, London, UK

The authors propose a systematic review and meta-analysis of Mendelian randomisation studies assaying the potential causal relationship between BMI and blood pressure traits. Great initiative and very much looking forward to the results! 

 Below some comment for consideration. 

 1.    Could the authors include some justification for focusing on BMI. There is increasing appreciation that additional anthropometric measurements may compliment BMI, i.e. reflecting sarcopenic obesity. Appreciate this is outcome specific of course, hence the question about justification. 

 2.    Regarding the meta-analysis, how likely is it that the MR studies source distinct GWAS data. I expect much of the underlying GWAS studies will have incorporated results from previous studies (e.g. cumulative GWAS), creating dependencies which may complicate naïve meta-analysis. How will the authors identify potential overlap? Perhaps good to provide a strategy regarding overlap and potential analytical solutions (including not doing a meta-analysis)? 

 3.    With that respect would a across genetic ancestry meta-analysis be relevant? Or does the author expect substantial difference in BMI effect on blood pressure? 

 4.    Table 1 – very much appreciate the sentiment in comparing IV and randomized trials. The assumption column seems a repetition for A and B, perhaps simply have A and B nested within assumption? 

 5.    Inclusion. How will the authors decide whether a study conducted a Mendelian randomisation analysis? Perhaps based on the estimator? 

 6.    Re the data collection. 

 a.    Many studies might not be fully one-sample or fully two-sample – i.e. partial overlap, how will this be recorded? Also, with regard to the planned analysis being stratified on this... 

 b.    Some of the categorical variables might be too restrictive (e.g. ancestry, MR methods, p-value adjustments method)

 c.    Beta is not an effect estimate type it is a letter of the Greek alphabet. Please define, perhaps “mean difference”? 

 7.    Regarding the meta-analysis, how will you deal with MR estimate that are per SD? Presumably the SD will differ per study? This might result in heterogeneity simply because of differences in unit. 

 8.    The author suggest using a fixed-effect meta-analysis estimator. Their justification for this seems to suggest that there would be no between study heterogeneity? I appreciate that MR estimators, just like most other estimators, make the implicit assumption about homogeneity. But not sure why MR here is different from other study designs? And what about difference due to technical artifacts, bias, MR estimators, or different genetic populations? Perhaps a bit more exposition here would make it clearer? 

 9. “We will perform a narrative synthesis for any BMI-blood pressure where a quantitative meta-analysis is not possible.” Please describe when this would be the case, a MA not being possible.

Is the study design appropriate for the research question?

Yes

Is the rationale for, and objectives of, the study clearly described?

Partly

Are sufficient details of the methods provided to allow replication by others?

Partly

Are the datasets clearly presented in a useable and accessible format?

Not applicable

Reviewer Expertise:

Epidemiology, Mendelian randomisation.

I confirm that I have read this submission and believe that I have an appropriate level of expertise to confirm that it is of an acceptable scientific standard.
